# Enhancing the Function of CD34^+^ Cells by Targeting Plasminogen Activator Inhibitor-1

**DOI:** 10.1371/journal.pone.0079067

**Published:** 2013-11-01

**Authors:** Sugata Hazra, Valerie Stepps, Ashay D. Bhatwadekar, Sergio Caballero, Michael E. Boulton, Paul J. Higgins, Elena V. Nikonova, Carl J. Pepine, Catherine Thut, Eva M. Finney, David J. Stone, Stephen H. Bartelmez, Maria B. Grant

**Affiliations:** 1 Department of Pharmacology and Therapeutics, University of Florida, Gainesville, Florida, United States of America; 2 BetaStem Therapeutics, Inc, San Francisco, California, United States of America; 3 Department of Anatomy and Cell Biology, University of Florida, Gainesville, Florida, United States of America; 4 Center for Cell Biology and Cancer Research, Albany Medical College, Albany, New York, United States of America; 5 Exploratory and Translational Sciences, Merck Research Laboratories, Merck & Co. Inc,West Point, Pennsylvania., United States of America; 6 Department of Medicine, University of Florida, Gainesville, Florida, United States of America; Cedars-Sinai Medical Center; UCLA School of Medicine, United States of America

## Abstract

Previously, we showed that transient inhibition of TGF- β1 resulted in correction of key aspects of diabetes-induced CD34^+^ cell dysfunction. In this report, we examine the effect of transient inhibition of plasminogen activator inhibitor-1 (PAI-1), a major gene target of TGF-β1 activation. Using gene array studies, we examined CD34^+^ cells isolated from a cohort of longstanding diabetic individuals, free of microvascular complications despite suboptimal glycemic control, and found that the cells exhibited reduced transcripts of both TGF-β1 and PAI-1 compared to age, sex, and degree of glycemic control-matched diabetic individuals with microvascular complications. CD34^+^ cells from diabetic subjects with microvascular complications consistently exhibited higher PAI-1 mRNA than age-matched non-diabetic controls. TGF- β1 phosphorodiamidate morpholino oligo (PMO) reduced PAI-1 mRNA in diabetic (p<0.01) and non-diabetic (p=0.05) CD34^+^ cells. To reduce PAI-1 in human CD34^+^ cells, we utilized PAI-1 siRNA, lentivirus expressing PAI-1 shRNA or PAI-1 PMO. We found that inhibition of PAI-1 promoted CD34^+^ cell proliferation and migration *in vitro*, likely through increased PI3(K) activity and increased cGMP production. Using a retinal ischemia reperfusion injury model in mice, we observed that recruitment of diabetic CD34^+^ cells to injured acellular retinal capillaries was greater after PAI-1-PMO treatment compared with control PMO-treated cells. Targeting PAI-1 offers a promising therapeutic strategy for restoring vascular reparative function in defective diabetic progenitors.

## Introduction

With the global pandemic of diabetes affecting every continent, the impact of diabetic micro- and macro-vascular complications is far reaching. Central to all vascular complications is endothelial dysfunction. However, equally significant is the inability to repair dysfunctional endothelium. The process of repair is mediated largely by vascular progenitor populations [1,2]. One such progenitor population, CD34^+^ cells are hematopoietic cells which exhibit altered *in vitro* and *in vivo* function in individuals with vascular complications [3-9]. 

CD34^+^ cells represent an ideal biomarker for the prediction of the cardiovascular disease, metabolic syndrome and type 2 diabetes [10]. CD34^+^ cells function to provide paracrine support to injured vasculature and tissues. Their reparative function has broad implications for supporting the health of an individual, and this has led to the use of these cells in clinical trials for treating ischemic conditions [11]. Transient downregulation and functional inhibition of the intracellular TGF-β1 pathway in diabetic human CD34^+^ cells corrects key aspects of their dysfunctional behavior [12] and this likely occurs through effects on critical TGF-β1 target genes. To this end, recent data confirms the role of one such TGF-β1-regulated gene, PAI-1 (SERPINE1), as an important mediator of cellular growth arrest [13]. PAI-1 is a single-chain glycoprotein (50 kDa molecular weight) that is present in the blood in very low concentrations in healthy subjects. PAI-1 blocks plasmin generation by inhibiting activities of serine proteinases, urokinase plasminogen activator (uPA) and tissue-type plasminogen activator (t-PA). Plasmin is a key enzyme in extracellular matrix (ECM) degradation. PAI-1 expression is influenced by specific cytokines and growth factors and its activity is regulated at the transcriptional level [14]. PAI-1 expression, like TGF-β, negatively regulates PI3K/Akt mediating cell survival, proliferation and migration [15-17]. Levels of PAI-1 are increased in the serum of subjects with obesity, diabetes and atherosclerosis [18]. Transcription of the PAI-1 gene is modulated by hypoxia [19]. Inhibition of PAI-1 using a PAI-1 selective antibody increased migration of human CD34^+^ across rat endothelial cell monolayer [20]. Moreover, the 4G/5G promoter allele of the PAI-1 gene is strongly linked to type 2 diabetes [21]. Increased levels of PAI-1 are accompanied by increased levels of urokinase and metalloprotease enzymes in human diabetic microvascular membranes [22]. PAI-1 expression is increased in retinas with oxygen-induced retinopathy [23]. Previously, we showed that PAI-1 is over expressed in the capillaries of diabetic individuals with non-proliferative diabetic retinopathy [24], and that PAI-1^-^/^-^ animals made diabetic are protected from the development of diabetic retinopathy [25]. CD34^+^ cells express low-density lipoprotein receptor-related protein 1(LRP-1), the putative receptor for PAI-1 [26], supporting that PAI-1 may mediate both paracrine and autocrine effects on CD34^+^ cells. 

We reasoned that the PAI-1 system could provide valuable insights into the function of CD34^+^ cells and, therefore, effective regulation of this system in diabetes might confer an enhanced reparative function of these cells and protection from the development of vascular complications. To test this hypothesis, we examined PAI-1 in CD34^+^ cells isolated from a unique cohort of diabetic individuals that, despite a lifetime of poor glycemic control, remained free of microvascular complications. We also studied the impact of normalizing high PAI-1 levels in dysfunctional CD34^+^ cells obtained from diabetic subjects with complications using *in vitro* and *in vivo* cell function. 

## Results

### Absence of an increase in PAI-1 in CD34^+^ cells in diabetic subjects predicted protection from the development of microvascular complications

**Figure 1:**
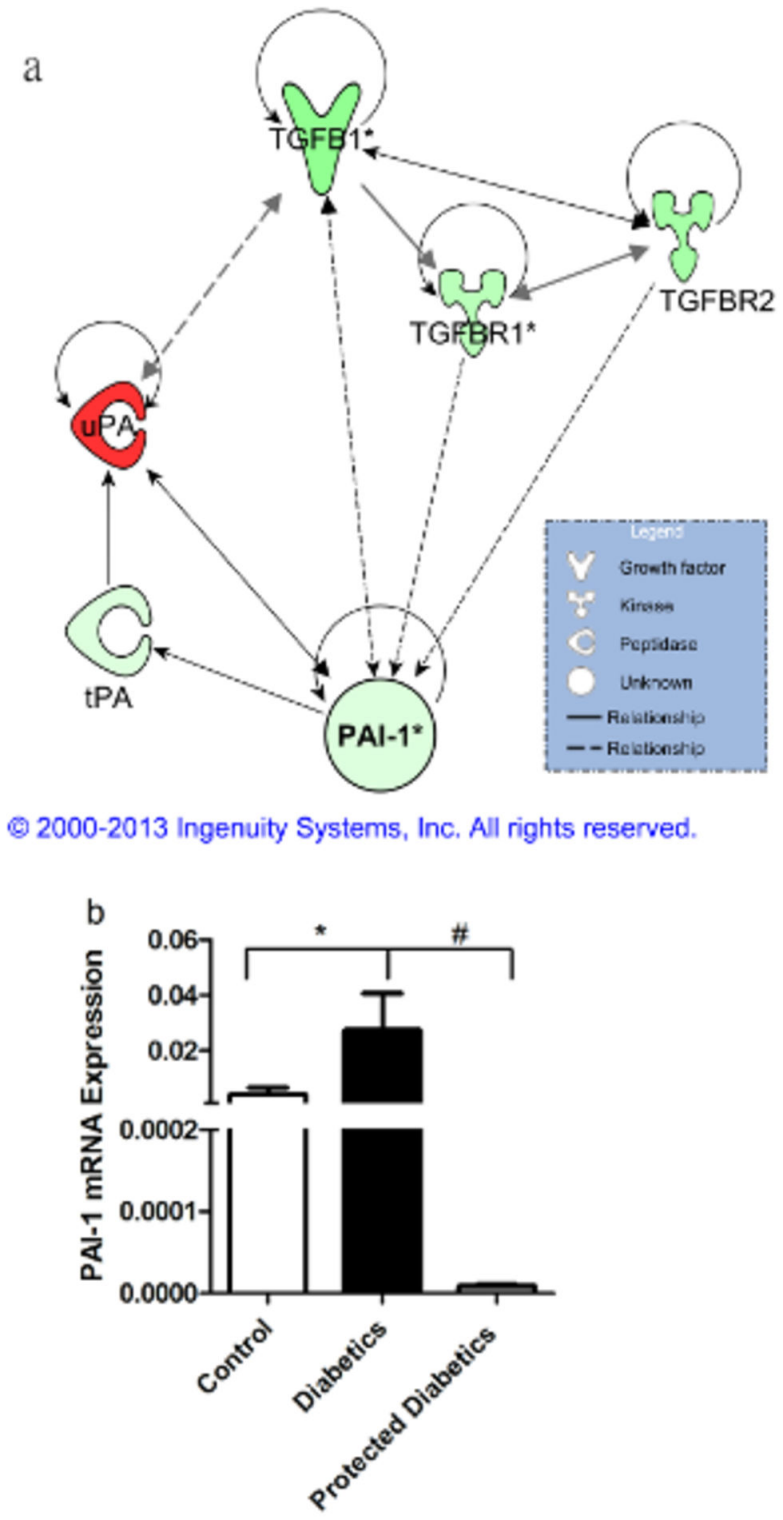
Diabetic individuals protected from development of microvascular complications exhibit reduced expression of PAI-1 and increased expression of uPA in CD34^+^ cells. Microarrays were conducted on CD34^+^ cells obtained from diabetic individuals with microvascular complications (n=5), diabetic individuals without microvascular complications (n=5), healthy age-matched controls (n=4). mRNA transcript levels in cells from protected diabetic individuals were compared to those from diabetic individuals with complications by t-test. 270 probe sets were found differentially expressed with p<0.001 (false discovery rate ~19%). (a) Ingenuity pathway map shows the gene expression profile. In CD34^+^ cells from protected individuals, TGF-β1, TGF-βR1, TGF-βR2, PAI-1 and tPA (tissue plasminogen activator) were downregulated whereas uPA (urokinase plasmonigen activator) was upregulated. (b) Microarray data was confirmed by real-time RT-PCR showing that protected individuals have low levels of PAI-1 mRNA compared to diabetic individuals.

We hypothesized that diabetic individuals protected from the development of microvascular complications might have more robust CD34^+^ cell function with a superior reparative response compared to CD34^+^ cells from diabetic individuals manifesting vascular complications. We identified a unique diabetic cohort without microvascular complications despite having diabetes for more than 40 years with largely poor metabolic control throughout this entire time. CD34^+^ cells from this cohort of protected subjects showed increased migratory potential compared to cells from diabetic subjects with microvascular complications [30]. Using gene array studies, we compared the CD34^+^ cells from protected diabetic individuals to diabetic individuals with microvascular complications that were matched for sex, age and glucose control, as well as to healthy controls using Affymetrix microarrays (Table 1). 270 probe sets were found differentially expressed with p<0.001 (false discovery rate ~19%) (Figure **1a**). Ingenuity pathway map shows the gene expression profile. In CD34^+^ cells from the protected individuals, TGF-β1, TGF-βR1, TGF-βR2, PAI-1 and tPA (tissue plasminogen activator) were downregulated whereas uPA (urokinase plasmonigen activator) was upregulated. We also measured PAI-1 expression in these three cohorts by real-time RT-PCR, which showed that protected individuals had low levels of PAI-1 in CD34^+^ cells compared to diabetic individuals (Figure **1b**). These results suggest that the “protected” subjects had reduced activation of the TGF-β1/PAI-1 system.

**Table 1:** Characteristics of subjects in cohort 1.

Donor Characteristics	Age	HbA_1C_ (%)	Duration of Diabetes
Healthy (5)	44.75±6.59	5.4±0.19	------------
Diabetic individuals without complications (5)	58.6±4.45	7.4±0.21	41.8±5.44
Diabetic individuals with complications (5)	56.2±2.28	7.9±0.4	27.4±5.77

### TGF-β1 inhibition reduces PAI-1 in stem/progenitors

**Figure 2:**
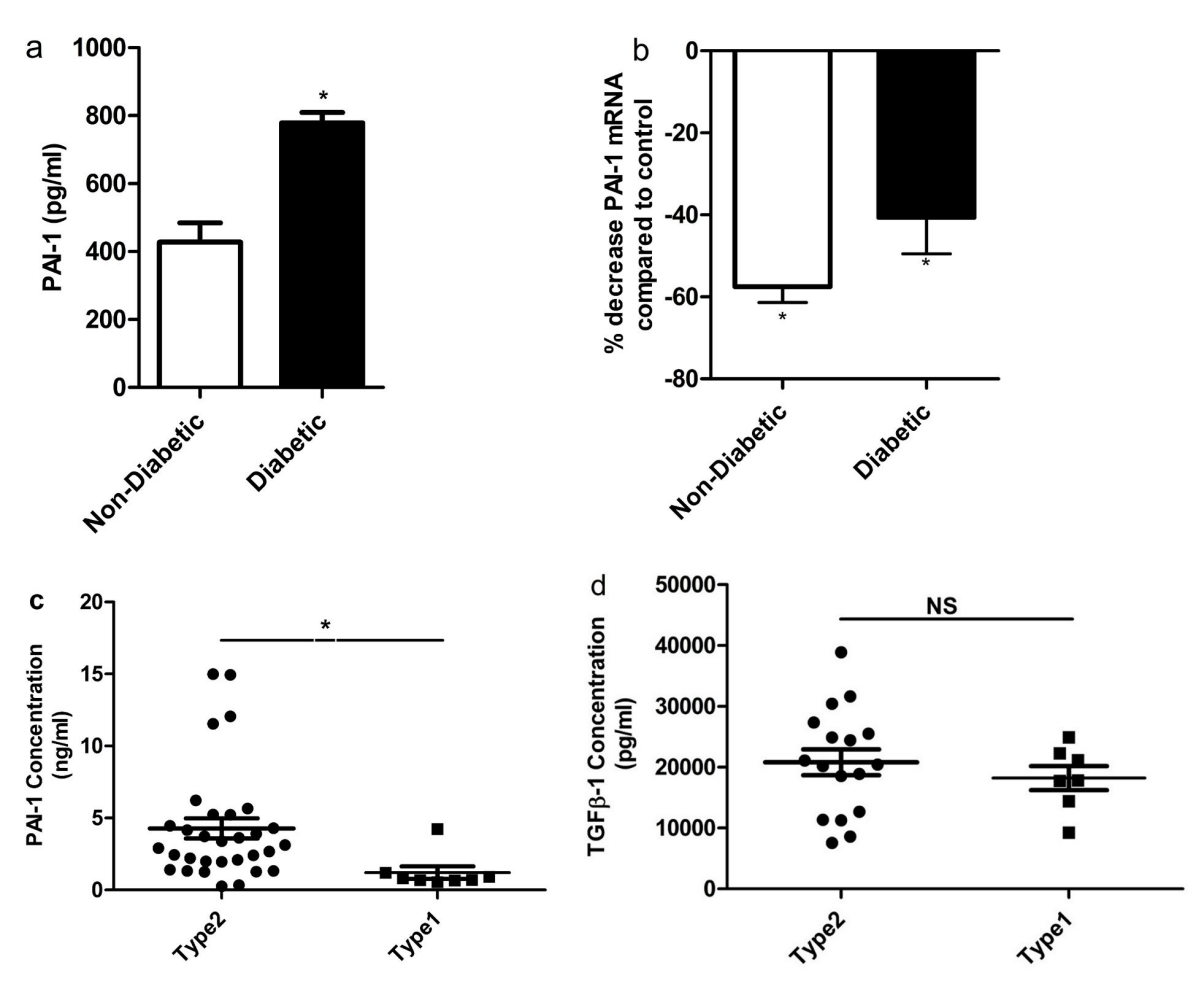
TGF-β1 mediates its action through PAI-1 in both diabetic and non-diabetic CD34^+^ cells. **(a)** PAI-1 concentration in the conditioned media of diabetic and non-diabetic CD34^+^ cells. A significant increase in the secreted level of PAI-1 was observed in the conditioned media obtained from diabetic CD34^+^ cells compared to non-diabetic (p<0.05) (mean ± SEM; n=3). (**b**) Effect of control PMO and TGF-β1PMO on PAI-1 gene expression in CD34^+^ cells was assessed. Both diabetic and non-diabetic CD34^+^ cells were pretreated overnight with either scrambled PMO or TGF-β1PMO (40ng/ml). PAI-1 mRNA transcripts were quantified by real-time RT-PCR and was normalized to β-actin levels. Values in cells treated with scrambled PMO were set at 1.0. p<0.001(for non-diabetic compared to scrambled PMO treated cells); p=0.05 (for diabetic compared to scrambled PMO treated cells); n=10 for diabetic and n=3 for control. (**c**-**d**) Plasma concentrations of PAI-1 and TGF-β1 in type1 and type2 diabetic individuals. A significant increase (p<0.05) in the plasma concentration of PAI-1 in type2 diabetic individuals compared to type 1 (n=31 for type 2; n=7 for type 1), although the concentration of TGF-β1 was similar in both groups (20815.1pg/ml in type2 and 18212.2 pg/ml in type1). Each dot represents one patient sample.

PAI-1 has been shown to be elevated in diabetes [31]. PAI-1 is central to various pathways that regulate cellular motility (e.g. uPA, TGF-β1), proliferation (e.g. ETS, MYC, AKT), and survival/stress (e.g. JNK, caspases, NFκB, TNFR) programs [32]. Since levels of PAI-1 increase in endothelial cells as a result of exposure to high glucose, high insulin, oxidative stress, or TGF-β1, we measured endogenous secretion of PAI-1 in CD34^+^ cells from individuals with diabetes. CD34^+^ cells of diabetic origin secreted significantly more PAI-1 into the CM compared to non-diabetic individuals (p<0.05) (Figure **2a**). To assess the impact of reducing TGF-β1 on endogenous PAI-1 mRNA expression, CD34^+^ cells of diabetic or non-diabetic origin were treated with either TGF-β1PMO or scrambled PMO prior to measurement of PAI-1 transcripts. Reduced PAI-1 mRNA levels were evident in both diabetic and non-diabetic CD34^+^ cells treated with TGF-β1 PMO compared to cells treated with scrambled PMO, (p<0.001 diabetic and p=0.05 non-diabetic) (Figure **2b**). TGF-β1 and PAI-1 secreted by circulating CD34^+^ cells contribute to plasma levels of these factors. Thus, we quantified plasma PAI-1 and TGF-β1 in type 2 subjects and compared the levels of the two proteins to those in type 1 subjects (Figures **2c, d**). Plasma PAI-1 levels were higher in type 2 diabetic individuals compared to type 1 diabetic individuals (n=31 for type 2, n=8 for type 1, p=0.03 Figure **2c**), while TGF-β1 levels were similar in both groups (n=17 for type2, n=7 for type1 Figure **2d**). These results combined with our finding that diabetic individuals protected from the development of microvascular complications exhibited lower PAI-1 levels in their CD34^+^ cells compared to subjects with complications, suggested to us that PAI-1 may represent a more viable target than TGF-β1 for the correction of CD34^+^ cell dysfunction in type 2 individuals. Thus, we asked whether inhibition of PAI-1 would have a beneficial effect on CD34^+^ cell function. 

### PAI-1 blockade stimulated growth of CD34^+^ cells

**Figure 3:**
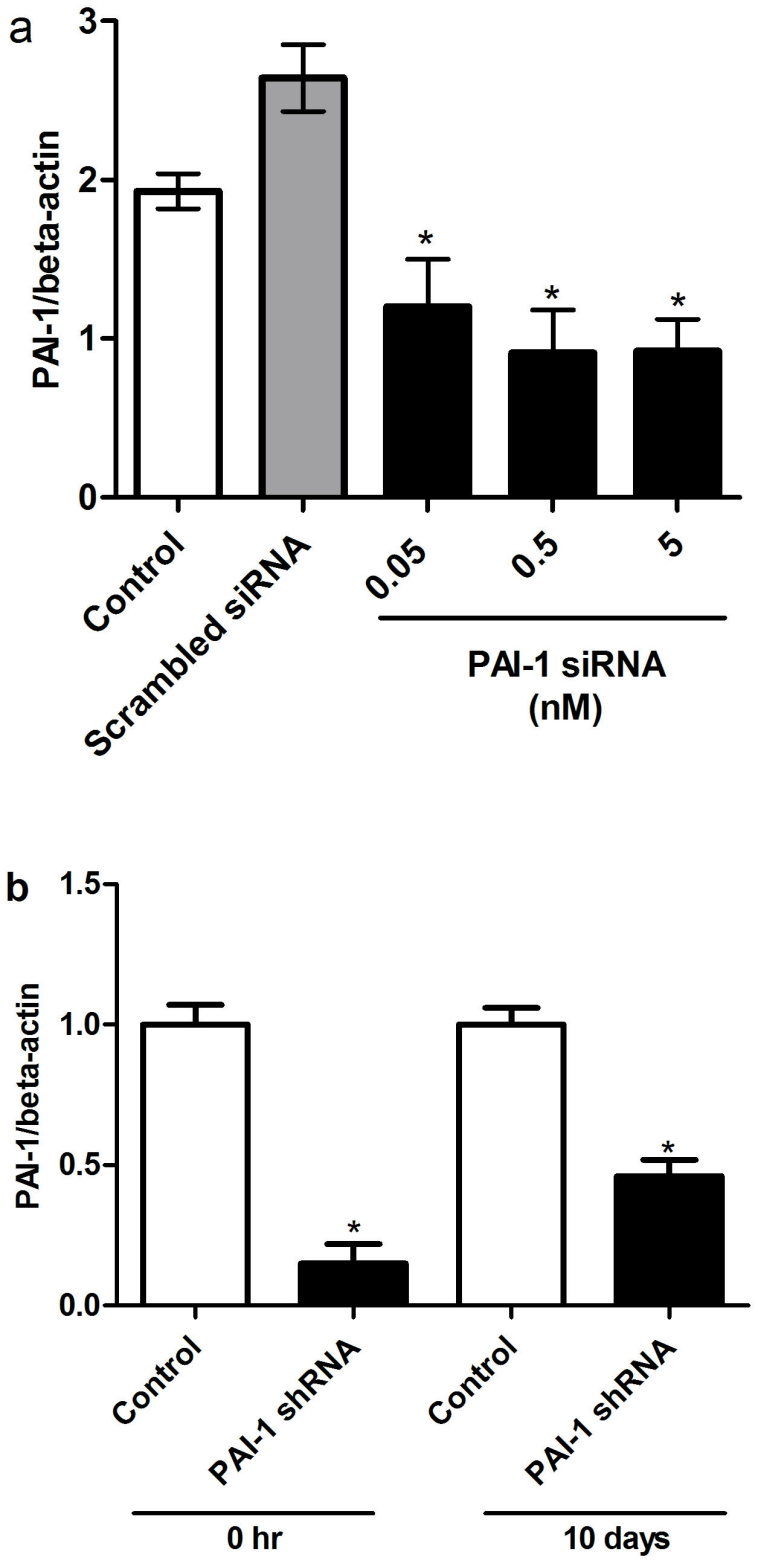
Inhibition of PAI-1 in CD34^+^ cells using PAI-1 siRNA and lentivirus expressing PAI-1 shRNA. RNA isolates were prepared from cell pellets followed by real-time RT-PCR for PAI-1. (**a**) CD34^+^ cells transfected with different concentrations of PAI-1 siRNA. (**b**) CD34^+^ cells transduced with lentivirus expressing PAI-1 shRNA and control immediately after infection (0hr) and 10days after from the colonies generated by CFC assay.*p<0.05.

**Figure 4:**
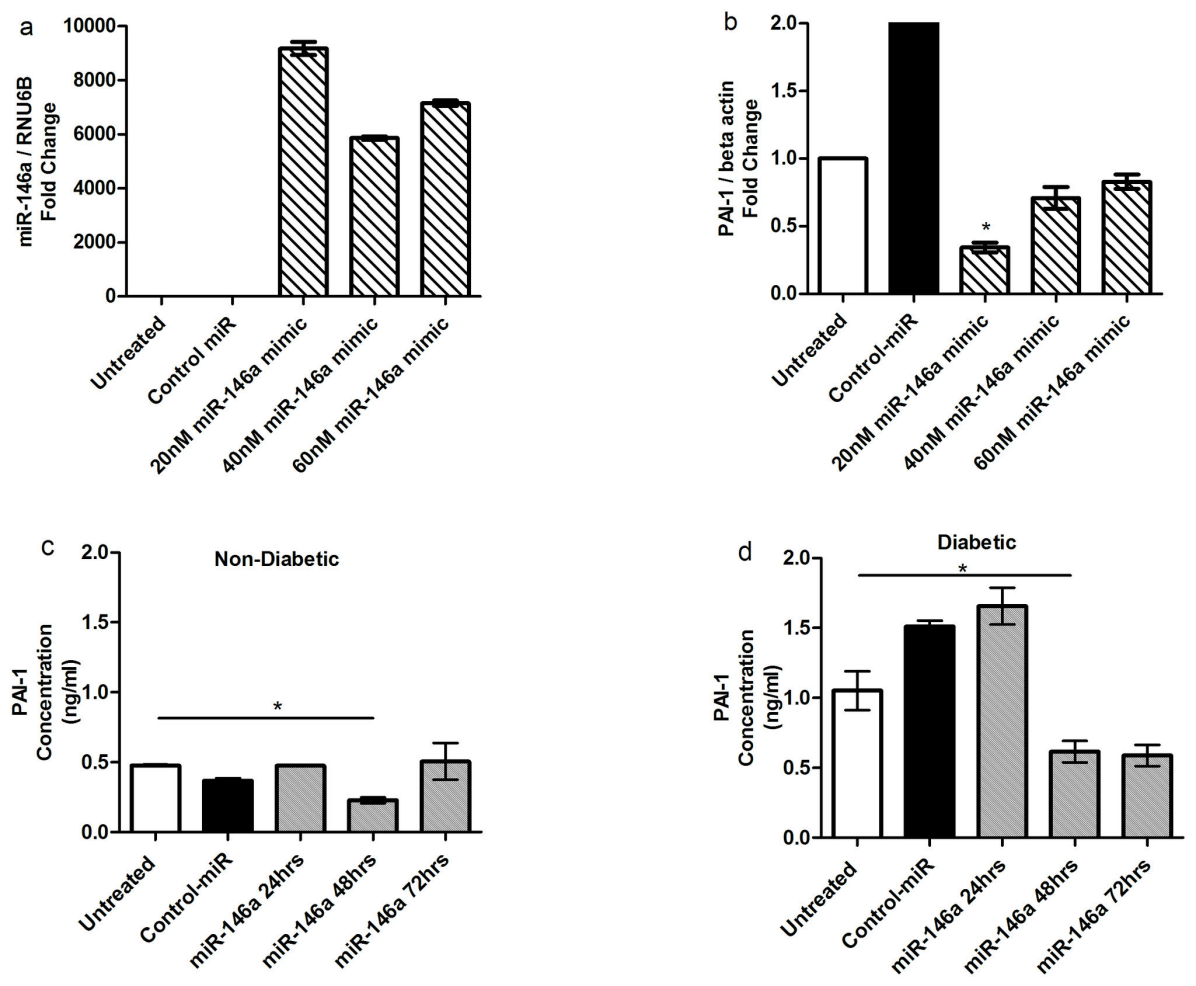
Inhibition of PAI-1 using miR-146a mimic. (**a**) Over expression of miR-146a mimic in CD34^+^ cells. CD34^+^ cells transfected with miR-146a mimic followed by incubation for 24hrs. RNA isolates were prepared from cell pellets followed by Real-time RT-PCR for miR-146a expression normalized to RNU6B. (**b**) Transfected miR-146a mimic decreases PAI-1 mRNA expression in CD34^+^ cells. Real-time RT-PCR for PAI-1 expression was normalized to β-actin. (**c**) Reduction of secreted PAI-1 in the conditioned media from non-diabetic CD34^+^ cells. (**d**) Reduced secreted PAI-1 in the conditioned media from diabetic CD34^+^ cells.

**Figure 5:**
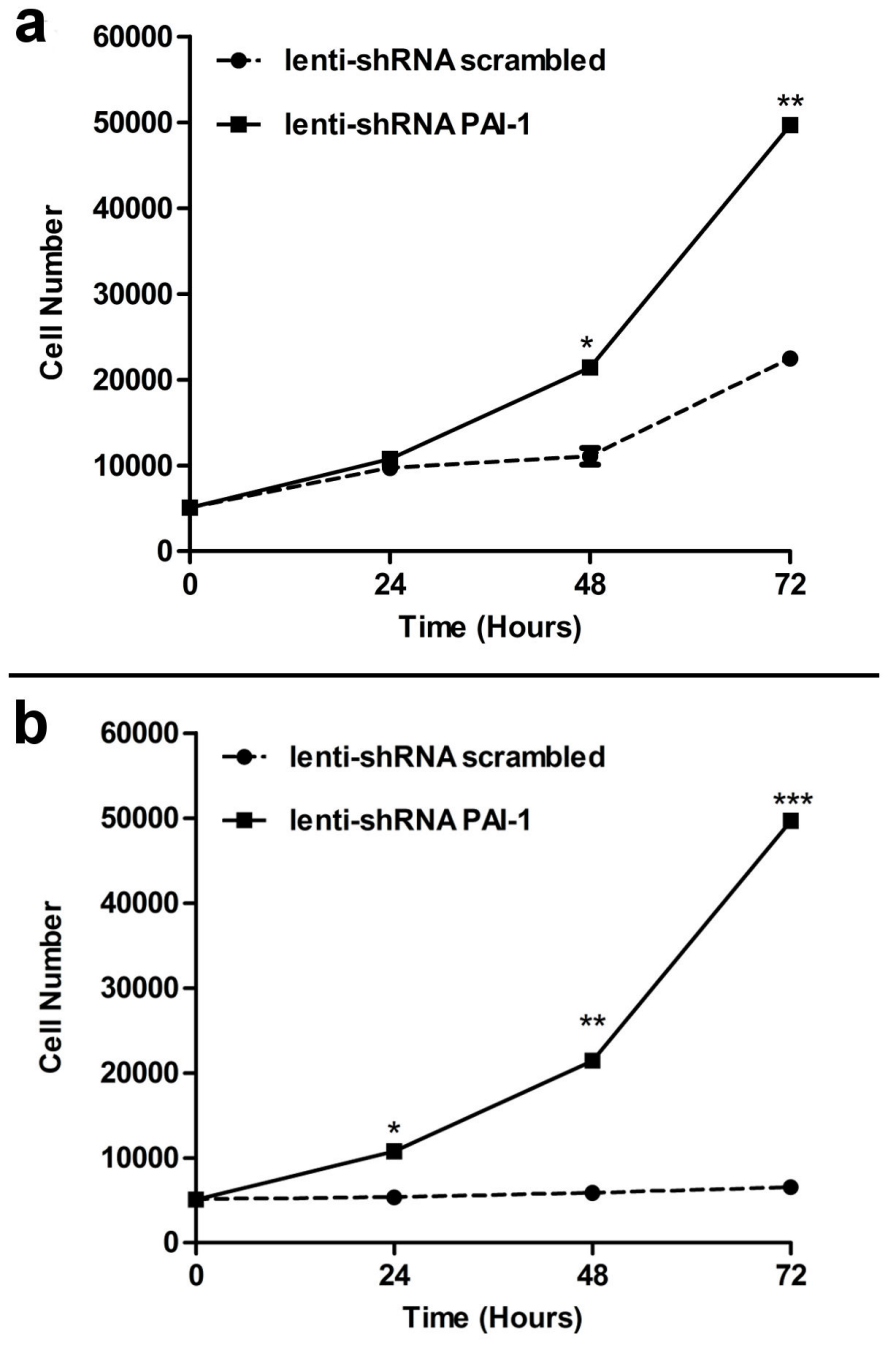
PAI-1 inhibition increased growth of healthy and diabetic CD34^+^ cells following 24 hr exposure. (**a**) Non-diabetic and (**b**) diabetic CD34^+^ cells were infected with either lentivirus expressing PAI-1 shRNA (solid line) or lentivirus expressing scrambled shRNA (broken line) for 2hrs and then cultured with added growth factors (cytokine cocktail) for up to 72 hrs. After every 24hr period, viable cells were counted using trypan blue. Each data point represents mean ± SEM for 3 separate experiments in duplicates.

 Three separate approaches were used to reduce PAI-1 in CD34^+^ cells; PAI-1 siRNA, lentivirus expressing PAI-1 shRNA (Figure **3**) and over-expressing miR-146a mimic (Figure **4**). All three approaches were efficient in reducing PAI-1 mRNA. In the presence of growth factors, inhibition of PAI-1 promoted cell growth in CD34^+^ cells of diabetic and non-diabetic origin (Figure **5a, b**). The growth of CD34^+^ cells of non-diabetic origin (Figure **5a**) following PAI-1 inhibition (solid line) was greater than cells treated with lenti shRNA control (dotted line). In contrast, the growth of CD34^+^ cells of diabetic origin did not improve (dotted line) even in the presence of growth factors; however, inhibition of PAI-1 remarkably increased the rate of growth of the cells of diabetic origin (solid line) to the level of non-diabetic cells (Figure **5b**). 

An important issue for cell therapy is the apparent need to expand CD34^+^ cells *ex vivo* in the absence of differentiation, prior to their re-introduction into individuals. To determine whether PAI-1 blockade could mediate such a therapeutically desirable effect, we assessed the number of cells in G_0_ and in G_1_ at days 5 and 7 at baseline conditions and following PAI-1 siRNA treatment. Following PAI-1 siRNA treatment, fewer cells were in G_0_suggesting that reducing PAI-1 facilitated the transition of cells through the cell cycle (data not shown). To minimize the exposure of progenitors to growth factors and thus to reduce the risk of differentiation and to reduce expansion, CD34^+^ cells were treated with PAI-1 siRNA in the presence of growth factors for only 24 hrs and then the growth factors were removed. Compared to control siRNA treated cells, inhibition of PAI-1 allowed a greater number of cells to survive in the absence of growth factors over 6 days (78.5% increase compared to control siRNA) (data not shown). 

**Figure 6:**
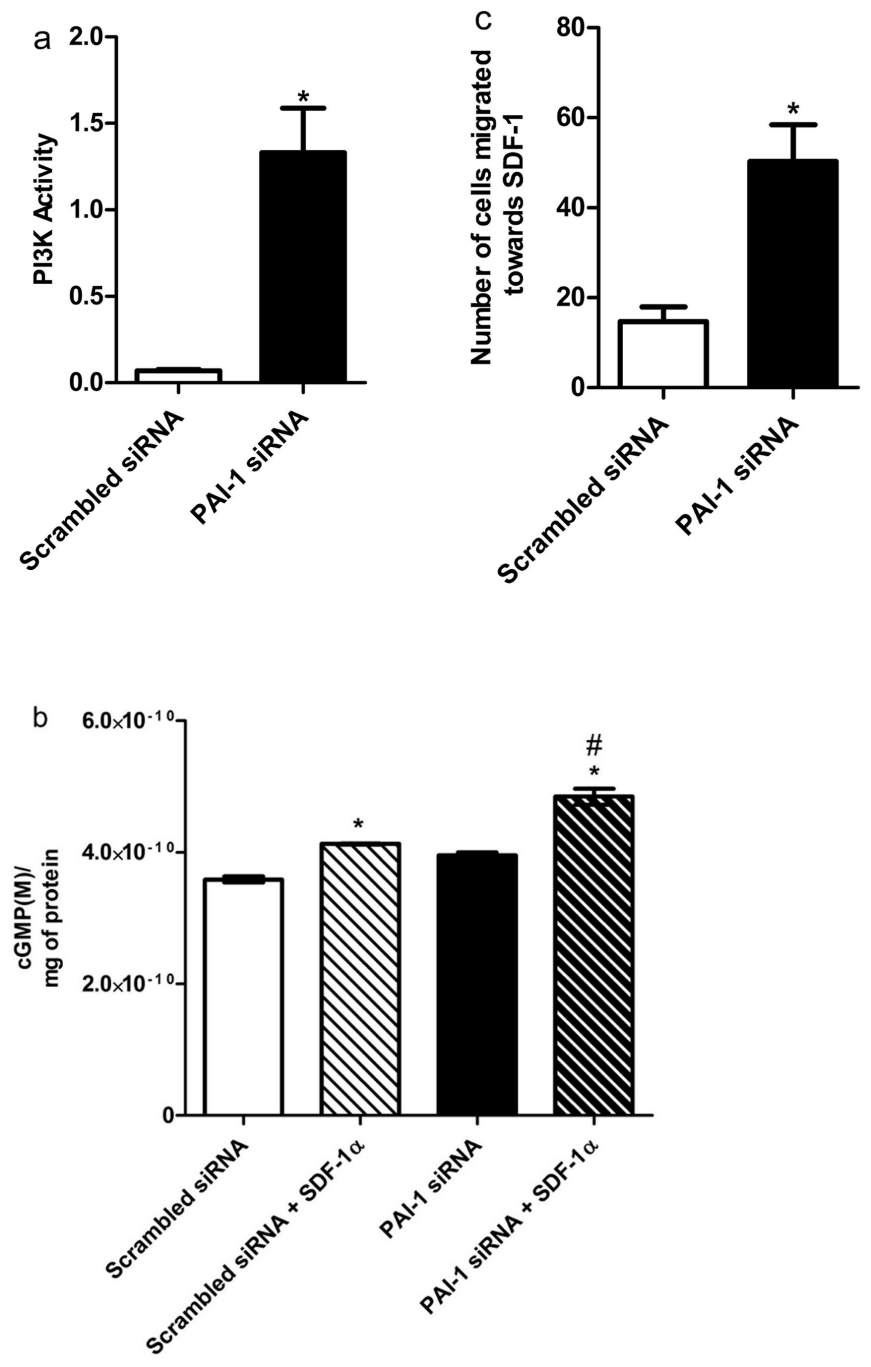
PAI-1 inhibition increases PI(3)K activity, cGMP production and improves migration. (**a**) PI3 kinase activity was measured in non-diabetic CD34^+^ cells by measuring the amount of PI(3–5) P_3_ produced from PI(4,5) P_2_ following PAI-1 inhibition. The amount of product produced was measured by ELISA. The bar graph is representative of 3 separate experiments. (**b**) cGMP production after PAI-1 inhibition was measured by chemiluminescence assay. (**c**) Boyden chamber assay showing migration of diabetic CD34^+^ cells to 100 nM of SDF-1α. Freshly isolated cells were exposed to either PAI-1 siRNA (5 nM) or scrambled siRNA and were then allowed to migrate towards SDF-1α (100 nM) for 18hrs. Numbers of migrated cells were counted. The graph shows the number of cells that migrated after being pre-exposed to either scrambled siRNA (black bar) or PAI-1 siRNA (white bar).

Studies on human adult fibroblasts indicated that PAI-1 knockdown leads to cell cycle progression by increasing phosphatidyl inositol 3-kinase (PI(3)K) signaling [13]. We asked whether this also occurred in CD34^+^ cells in which PAI-1 was reduced. The effect of inhibition of PAI-1 on PI3K activity in CD34^+^ progenitors was tested using the conversion of PI(3–5)P_3_ to PI(4,5)P_2_. Blocking PAI-1 resulted in stimulation of PI(3)K activity compared to scrambled siRNA treatment (p<0.05) (Figure **6a**) in CD34^+^ progenitors.

### PAI-1 inhibition improved migration of CD34^+^ cells of diabetic origin

Critical to the function of CD34^+^ cells is their ability to migrate to areas of injury and support vascular repair. Bioavailable NO within these cells is critical for their ability to home and migrate [33]. CD34^+^ cells of diabetic origin demonstrate reduced NO bioavailability [34]. As PI(3)K-AKT signaling is central to eNOS expression, we next determined whether inhibition of PAI-1 was associated with increased cGMP production needed for NO generation. In CD34^+^ cells of diabetic origin, inhibition of PAI-1 increased cGMP production both in basal condition and after SDF-1α(100nM/L) stimulation by 10% and 17% respectively (Figure **6b**). Moreover, PAI-1 inhibition improved the migratory response of CD34^+^ cells of diabetic origin to SDF-1α compared to cells treated with scrambled siRNA (Figure **6c**), suggesting that reducing PAI-1 towards normal non-diabetic levels corrected the migratory dysfunction of diabetic cells *in vitro*. 

### PAI-1 PMO treatment of CD34^+^cells enhances in vivo reparative function of cells in the I/R injury model

**Figure 7:**
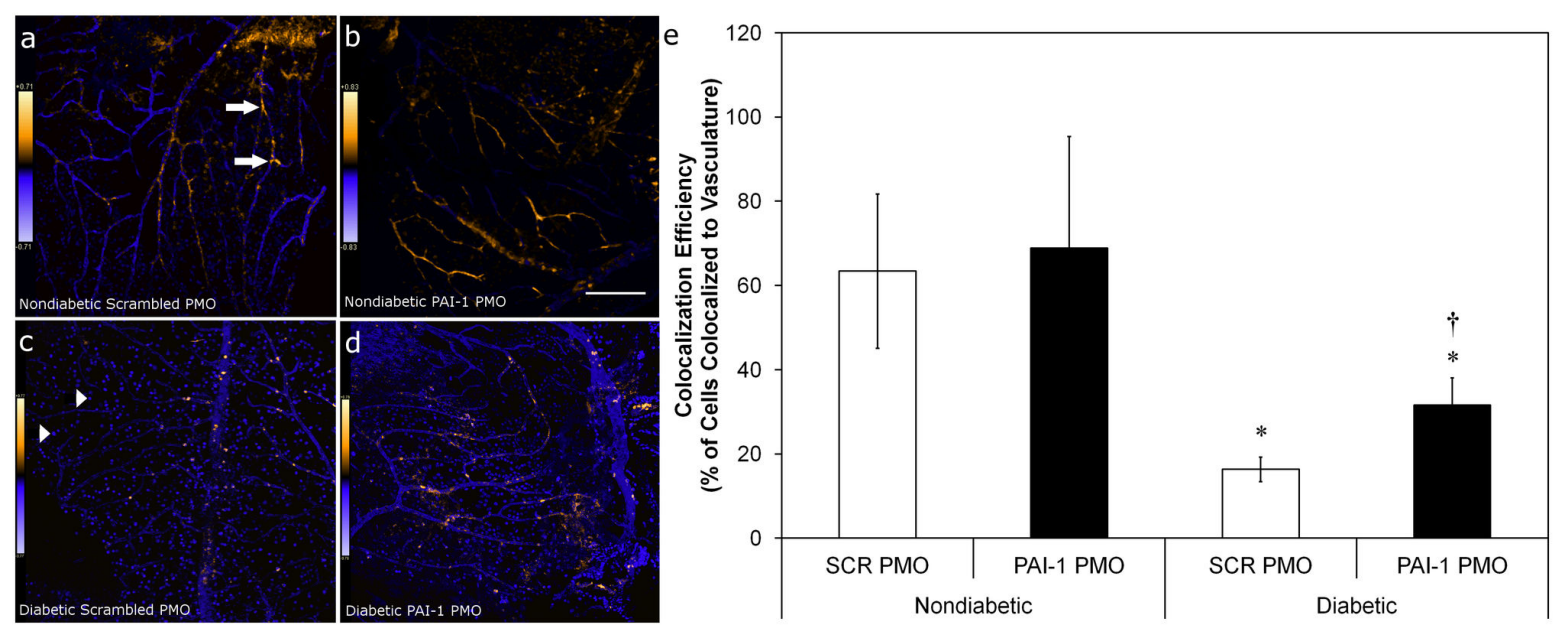
PAI-1 inhibition with PMO increases diabetic CD34^+^ cell homing to and association with vasculature in an acute I/R model. CD34^+^ cells isolated from diabetic (n=8) or age- and sex-matched normal (n=4) donors were exposed to either scrambled (SCR) or PAI-1-specific PMO for 16hr prior to intravitreous injection 7 days post ischemic insult. Eyes were harvested 2 days later and processed for immunofluorescence and data was collected using laser scanning confocal microscopy. Panels (**a**-**d**) are typical fields that show calculated co-localization of CD34^+^ cells with retinal vasculature using Intensity Correlation Analysis (Mander’s coefiicient) and given false color to indicate the degree of co-localization probability (warmer colors = higher probability). Scale bar in (**b**) also applies to (**a**) and is 100 mm. Scale bar in (**d**) also applies to (**c**) and is 150 mm. (**e**) represents co-localization efficiency. Arrows indicate exogenously added cells that have not co-localized to vasculature (best seen in Panel C), while arrowheads (Panel A) indicate areas where exogenously added cells co-localize strongly (warm colors) with vessels. * = p<0.05 vs. non-diabetic cells with the same pre-treatment. † = p<0.05 versus SCR PMO with diabetic cells.

The *in vivo* vasoreparative function of PAI-1 PMO modified CD34^+^ cells was evaluated using a mouse model of I/R injury that recapitulates many features of diabetic retinopathy, including the presence of acellular capillaries [35]. Cells were injected intravitreally into injured eyes, and the homing of cells to areas of injury, a direct indicator of the *in vivo* migratory prowess of these cells, was expressed as percent of the total vascular area. Previously, we showed that cells of diabetic origin display markedly reduced homing to areas of injury. Cells of diabetic origin form aggregates on the surface of the vitreous and do not associate with the retinal vasculature [29,36]. In the I/R model of acute retinal vascular injury, between 60-70% of detected CD34^+^ cells from healthy donors home to and associate with vasculature [29,36]. No difference was measured in association of CD34^+^ cells of non-diabetic origin with vasculature in cells pre-treated with either scrambled PMO or cells pretreated with PAI-1 PMO. By contrast, CD34^+^ cells from diabetic donors pre-treated with scrambled PMO exhibited poor homing and association with vasculature, with less than 20% of detected cells co-localizing with vessels. In contrast, when these CD34^+^ cells were treated with PAI-1 PMO they showed a marked increase in co-localization with injured retinal vasculature (Figure **7**) (p<0.05). 

## Discussion

CD34^+^ cells were selected for the study as this population represents a good marker for metabolic disorders. The use of a miltenyi device for the isolation of these cells is approved by the FDA for human clinical trials and is fully approved in Europe. Autologous CD34^+^ cells hold promise to prevent tissue damage and restore blood flow in diabetic individuals or individuals with metabolic syndrome who may not be ideal candidates for standard revascularization procedures due to a diffuse vascular disease or failed previous revascularization. However, the dysfunctional biology of these cells in diabetes (the reduced proliferation, migration, and differentiation into endothelial cells [7,37]) limits their therapeutic utility [5,9,38,39]. 

Our focus on PAI-1 arose from the observation that diabetic individuals protected from vascular complications despite less than optimal diabetes control showed lower PAI-1 transcript levels in their CD34^+^ cells, and these same individuals expressed higher levels of uPA. uPA, much like NO, is needed to promote cell migration [26], which is a major function of these cells as they need to home to areas of injury to facilitate repair. CD34^+^ cells isolated from diabetic individuals with vascular complications show reduced NO bioavailability [12,40], and this decrease in NO is associated with reduced migration that can be corrected through exposure to NO donors [7]. The latter finding supported the notion that restoration of autologous CD34^+^ cell function in type 2 diabetic individuals represented a reasonable therapeutic option versus substitution of healthy allogeneic cells.

PAI-1 may provide a more efficacious and potentially safer target than TGF-β1, as PAI-1 has a narrower range of effects. Pre-treatment of CD34^+^ cells with PAI-1 siRNA, shPAI-1 lentiviruses or miR-146a, reduced PAI-1 mRNA and protein levels, which resulted in enhanced growth and migration *in vitro*. PAI-1 inhibition induced G_0_ exit and entry into the pre-cycling G_1_ state, reversing the profound cell cycle arrest observed in diabetic progenitors [6]. We also showed that if PAI-1 is inhibited in diabetic CD34^+^ cells: i) cells proliferated faster following one day of growth factor exposure; ii) subsequent growth factor withdrawal did not result in cell death; and iii) CD34^+^ cells from type 2 diabetic individuals survived for greater than a week *ex vivo* in the absence of growth factors. PAI-1 inhibition in CD34^+^ cells was also associated with increased PI3K activity, reflective of both their improved viability and migratory response. PI3K activation and subsequent Akt pathway engagement results in eNOS activation by phosphorylation at Ser1177, and leads to NO generation necessary for effective cell migration [41]. Most importantly, we tested the effect of inhibition of PAI-1 *in vivo* using PAI-1 PMOs in type 2 diabetic CD34^+^ cells. Since individuals with type 2 diabetes have CD34^+^ cells expressing high levels of PAI-1, their CD34^+^ cells theoretically will benefit from having levels of PAI-1 reduced toward a normal, non-diabetic range prior to the use of these cells as an autologous cell therapy. 

In conclusion, inhibition of PAI-1 in CD34^+^ cells in type 2 diabetic individuals enhances their *in vitro* and *in vivo* function. While an attempt is being made to replace traditional approaches for alleviating tissue ischemia (e.g., stents, angioplasty, or vessel grafts) with cell therapy, autologous cell therapy is limited in type 2 diabetic individuals because of dysfunctional cells. In CD34^+^ cells that express high levels of PAI-1, transient reduction of this factor towards the normal range may represent a promising therapeutic strategy to restore vascular reparative function in diabetic CD34^+^ cells.

## Materials and Methods

### Patient selection and characterization

Peripheral blood was collected from both type 1(n=8) and type 2 (n=58) diabetic individuals as well as from sex- and age-matched healthy controls (n=26). This study was conducted under Institutional Review Board of University of Florida (IRB) approval # IRB 570-2008. Participants gave written informed consent to participate in this study and Declaration of Helsinki protocols were followed. Individuals having HIV, Hepatitis B or C, ongoing malignancy, current pregnancy or history of organ transplantation were excluded from this study. Pertinent characteristics of the individuals are described in Table 1. A second cohort of subjects was used in this study that was protected from vascular complications despite having long-standing, poorly-controlled diabetes, and was compared to subjects of similar age and glycemic control but with microvascular complications (Table 2), as well as to non-diabetic subjects. 

**Table 2:** Characteristics of the subjects in cohort 2.

Patient Characteristics	Healthy	Type 1 Diabetic	Type 2 Diabetic
Number (N)	26	8	58
Age (years)	54±3.32	40±5.3	57±1.52
Duration of Diabetes (years)	---------	13±3.15	14±1.41
	**Clinical**		
Smoking (n)		2	14
Hypertension (n)		4	32
Diabetic Retinopathy (n)		1	8
Diabetic Neuropathy (n)		1	12
Diabetic Nephropathy (n)		3	2
**Metabolic Data**			
HbA_1C_	---------	7.6±0.8%	8.4±0.27%
	**Medications**		
Oral Hypoglycemic			
Metformin (n)	----------		7
Sulfonylureas (n)	----------		4
Combinations			
Aspirin (n)			1
Statins (n)			7

### Microarray analysis

RNA from CD34^+^ cells was extracted using Trizol, followed by AffyNugen amplification and cDNA was hybridized to Human RSTA Affymetrix 2.0 chips using ultra-low input protocol. After robust multi-array average (RMA) normalization [27], analysis of the data was performed using one way ANOVA comparing diabetic individuals with and without microvascular complications and comparing diabetic individuals to controls. Changes in gene expression were further analyzed through the use of Ingenuity Pathways Analysis software version X (Ingenuity® Systems, http://www.ingenuity.com/). 

### Isolation of Human CD34^+^ Cells from Peripheral Blood of Diabetic and Healthy Individuals

Blood was collected from individuals using cell preparation tubes (CPT) with heparin (BD Biosciences, San Jose, CA). After density gradient centrifugation at room temperature in a swinging bucket rotor for 30 mins at 2,200 rpm, the buffy coat containing leukocytes was collected. RBC contamination was removed using ammonium chloride solution (Stem Cell Technologies, Vancouver, Canada). Mononuclear cells were enriched for CD34^+^ cells by positive selection using human CD34^+^ cell enrichment kit (Stem Cell Technologies). In selected studies, CD34^+^ cells were maintained in culture in Stem Span^TM^ media (Stem Cell Technologies) supplemented with Stem Span^TM^ CC100 cytokine cocktail (Stem Cell Technologies).

### Collection and analysis of conditioned media

CD34^+^ cells (30,000 cells/ well) were incubated with 100 μl Stem Span^TM^ media with Stem Span^TM^ CC100 cytokine cocktail (Stem Cell Technologies) and antibiotics for 24 hrs, yielding conditioned media (CM). The CM was collected for the analysis of PAI-1 protein. An ELISA kit (Quantikine, R&D Systems, Minneapolis, USA) was used to quantify PAI-1 in the CM. PAI-1 values was expressed as pg per 30,000 cells. 

### Ex vivo pre-treatment of CD34^+^ cells using phosphorodiamidate morpholino oligomers (PMO) to TGF-β1

CD34^+^ cells isolated from normal and diabetic subjects were pretreated with 40µg/ml of either scrambled PMO or TGF-β1PMO overnight at 37°C in Stem Span^TM^ (Stem Cell Technologies) as previously described [12]. 

### Real time RT-PCR

1μg of total RNA was extracted from the CD34^+^cells with Trizol and was reverse-transcribed using an iScript cDNA synthesis kit (Bio-Rad, Hercules,CA) according to manufacturer’s protocol. Real-time RT-PCR was performed using ABI TaqMan protocol (ABI Biosystems, Foster City, CA). FAM-labeled primer for PAI-1 was used (ABI Biosystems). All samples were normalized to β-actin (ABI Biosystems). 20ul PCR reactions were assembled on 96-well plates and conducted using ABI 7500 Fast PCR system for 40 cycles with default thermal cycler parameters. A complete list of TaqMan assay IDs can be found in Table 3. Fold-change calculations were performed using 2^-ΔΔCt^ method [28].

**Table 3:** Human primers used for real-time RT-PCR.

Gene Symbol	Identification Number (Applied Biosystems)
PAI-1	Hs01126606_m1
ACTB (β-actin)	Hs99999903_m1
hsa-miR-146a	000468
RNU6B	001093

### Analysis of plasma PAI-1 and TGF-β1

Blood was collected in EDTA tubes and centrifuged at 1000g for 15mins to separate plasma. A 50μl sample from each donor was analyzed by sandwich enzyme linked immune sorbent assay (ELISA) using a commercially available assay kit (Quantikine, R&D Systems Inc.).

### CD34^+^ cell infection with lentivirus

Lentivirus expressing PAI-1 shRNA or scrambled shRNA were prepared as follows. The SERPINE1 (PAI-1) shRNA target sequence is TCTGTACAAGGAGCTCAT. Cloning was performed into the pLKO.1 (Addgene) lenti vector and lentiviruses were generated by transfection into HEK-293T cells expressing envelope (pMD2.G) and packaging (psPAX2) plasmids (standard Addgene protocol). The CD34^+^ cells were centrifuged at 300g for 5mins and supernatant was removed. The cell pellet was resuspended in DMEM (high glucose) supplemented with polybreen (10μg/ml) and 10% FBS to a final concentration of 5X10^4^ cells/ml. Cells were then infected with lentivirus expressing non-specific shRNA or lentivirus expressing PAI-1 shRNA with a multiplicity of infection of ~35. Cells were centrifuged at 23°C at 150g for 2hrs. After infection, cells were washed with PBS and cultured in Stem Span^TM^ (with/without added growth factors for the desired time period). 

### Cell viability assay

Cell viability was assessed using either trypan blue exclusion, where cells that excluded the dye were counted using a hemocytometer or using propidium iodide exclusion as detected using an LSRII flow cytometer analyzer.

### Cell cycle analysis

A stock solution of HØ dye (DNA intercalator) was freshly thawed and serially diluted with warm IMDM+10% FBS. Each cell sample was resuspended in 50-100µL of media (either IMDM+10% FBS or culture medium for the sample condition), and the cell suspension was added to the HØ. Cells were placed at 37°C to incubate for 1hr, protected from light. Twenty mins later, cells were removed briefly from the incubator and Pyronin Y (mRNA detector) was added. Cells were gently mixed and placed back into the incubator for 40min. One hour post HØ exposure, samples were pelleted, supernatant was aspirated and cold blocking buffer was added. After 10min of incubation at 4°C in the dark, the desired surface antibodies were added and allowed to incubate for a minimum of 20min. Cells were washed with FACS buffer, then resuspended in an appropriate amount of the same buffer and stored at 4°C in the dark until FACS acquisition. Single color compensation controls for each mouse monoclonal antibody were made using the BD^TM^ CompBeads kit according to manufacturer’s recommendation (BD Biosciences, San Jose, CA). Two aliquots of cells were stained either with HØ only or with Pyronin Y to create the nucleic acid dye compensation controls.

### CD34^+^ cell transfection with miRNA mimic

 Pre-miR miRNA precursor molecules (miR-146a mimic) were purchased from Ambion, dissolved into nuclease-free water and the resulting 50μM stock was stored in aliquots at -80°C. CD34^+^ cells (6X10^3^ cells/ well) were transfected with 20nM, 40nM, or 60nM of precursor or negative control using Lipofectamine 2000 (Invitrogen, Grand Island, NY) according to manufacturer’s instructions. CD34^+^ cells transfected with miR-146a mimic were incubated for 24hrs, and cell supernatants were collected for the measurement of PAI-1 secretion. Cell pellets were used for RNA isolation and real-time RT-PCR analysis.

### Quantification of miRNA and mRNA expression level by real-time RT-PCR

Total RNA from CD34^+^ cells was isolated using Trizol reagent (Invitrogen) following the manufacturer’s protocol. RNA concentrations were determined using NanoDrop ND-1000 spectrophotometer (NanoDrop Technology Inc, Wilmington, DE). miRNA analysis was done using the TaqMan MicroRNA Reverse Transcription Kit, TaqMan Universal PCR Master Mix and TaqMan MicroRNA Assay Primers for human miRNAs (Applied Biosystems, Foster City, CA). For mRNA analysis, iScript cDNA synthesis kit (Biorad) and Taqman mRNA assay primers for PAI-1 were used. Cycle threshold values (Ct) corresponding to the PCR cycle number at which fluorescence emission reaches a threshold above baseline emission were determined and miRNA expression values calculated using RNU6B as an endogenous control following the 2^-ΔΔCt^ method. After normalization to β-actin, mRNA expression values were quantified in the same way.

### siRNA transfection

Freshly isolated CD34^+^ cells were transfected with scrambled siRNA or PAI-1 siRNA using lipofectamine (Invitrogen) as the transfecting reagent. Opti-MEM I reduced serum medium was used as the transfection medium. Transfection was performed as per manufacturer’s instructions (Invitrogen).

### Cell migration of CD34^+^ cells using Boyden chamber assay

Cell migration was performed using the Boyden chamber assay. Briefly, cells were suspended in EBM-2 media and 10,000 cells were placed per well. Wells were covered with 5-µm pore membrane coated with type1collagen. The assembled chamber was inverted and placed for 2 hours at 5% CO_2_ to allow cell attachment to the membrane. Chambers were placed right side up and 100nM of the chemo-attractant SDF-1α was added to the top chamber, which was placed inside the incubator for 18hrs. Chambers were disassembled, adhered cells were scraped from the surface and the membrane was fixed and stained. Only cells that had migrated through the membrane were counted.

### PI3 kinase activity assay

Activation of PI3 Kinase by blocking PAI-1 was evaluated by measuring PI (3–5) P_3_ synthesis in CD34^+^ cells using PI(4,5)P_2_ as a substrate. Briefly, cell suspension was incubated with either scrambled siRNA or PAI-1 siRNA. Following incubation, the cells were lysed with lysis buffer. The lysate was collected and the protein concentration was measured using BCA Protein Assay (Thermo Scientific, Rockford, IL). Lysates were incubated with anti-PI3 kinase antibody (Upstate Biotechnology, Billerica, MA) at 4°C overnight, followed by addition of the 50% Protein A-agarose beads (Santacruz Biotechnology, Santa Cruz, CA). Immunoprecipitates were washed with a wash buffer and immunoprecipitated enzyme was added to the wells of a 96-well microplate, coated with PI(4,5)P_2_. ELISA was performed according to manufacturer’s instructions (Echelon Biosciences, Salt Lake City, UT). Enzyme activity was expressed as amount of PI(3–5)P_3_ produced/µg of cell protein. 

### Measurement of cGMP production

cGMP production in response to SDF-1α (100nM/L) was measured using HitHunter cGMP assay kit (DiscoverR_x_ Corporation, Fremont, CA) according to manufacturer’s instructions. Briefly, 20,000 cells were used per treatment. The cells were treated with SDF-1α for 4hrs and cGMP production was compared between un-stimulated and stimulated cells. The luminescence was measured by a plate reader (Biotek Instruments, Winooski, VT).

### Cell survival assay

Cells were treated with PAI-1 siRNA as described above, and cell cultures were observed and counted on day 5 and day 7. The cells were exposed to growth factors for a period of 24 hr, after which the growth factors were removed and the cells remained without any added growth factors for the rest of the culture period.

### Retinal ischemia-reperfusion (I/R) injury model

The animal study was approved by the institutional animal care and use committee (IACUC) at the University of Florida, and studies were conducted in accordance with the principles described in the Association for Research in Vision and Ophthalmology Statement for the Use of Animals in Ophthalmic and Vision Research. Mice were purchased from Jackson Laboratory. The ischemia/reperfusion (I/R) injury was performed as described previously [29]. No steps to ameliorate suffering are necessary with the approved I/R protocol as it is a minor puncture with a very narrow gauge needle. At study termination, the animals were killed by overdose of ketamine and xylazine (14 and 30 mg/kg, respectively) followed by thoracotomy, at which time the eyes were removed for immunonohistochemical processing. 

### Statistics

Student t-tests were conducted for group comparisons with p-value below 0.05 for significance, and One-Way ANOVA followed by Tukey’s post-hoc test were used for multiple comparisons. 
